# Supplementary material for: Key risk factors for substance use among female sex workers in Soweto and Klerksdorp, South Africa: A cross-sectional study
Source: PLoS One. 2022 Jan 21;17(1):e0261855. doi: 10.1371/journal.pone.0261855 (PMC8782394; doi:10.1371/journal.pone.0261855)
Supplement: S1 Questionnaire — (PDF) [file pone.0261855.s001.pdf]

Screening Tool

Please enter the coupon number that the participant enters with, if applicable	______________________
Select the coupon number that the participant enters with from the pack combinations<br>You can type the number and then choose from the auto-complete list	______________________
Date of Screening<br><br>______________________	
Biological gender (what you were born)	◯ Female ◯ Male ◯ Other
Self-identifies as a SW, or self-identifies as working within a SW hotspot and consensually trading in sex for money or goods as her primary source of income (PAST 6 MONTHS)?	◯ Yes ◯ No
Working (as a sex worker) in Matlosana area?	◯ Yes ◯ No
Age (if DoB unknown)	______________________
Current victim of human trafficking or sexual exploitation?	◯ Yes ◯ No
Do you believe this person is a sex worker?	◯ Yes<br>◯ Yes, only after secondary screening<br>◯ No, after secondary screening.
SUCCESSFULLY SCREENED! :)<br>Please enroll this person	
HIV status known or unknown	◯ Known ◯ Unknown
How many female sex workers do you know in Klerksdorp? By 'know' I mean people you probably know by name but you would greet if you saw them, and they would greet you back.	______________________<br>(IF: >250 then comment on actual amount and insert 250 in the answer field)
Of those female sex workers, about how many are over the age of 18 years?	______________________
Of those female sex workers that you know, how many have you seen over the past month?	______________________

How do you know the person who gave you this coupon?	◯ Friend<br>◯ Family<br>◯ Someone I see sometimes<br>◯ Stranger/Unknown person<br>◯ seed
Was the coupon given to you or was it brought/traded?	◯ Given ◯ Brought /traded

Preliminary

Consented to both questionnaire and HIV/TB tests	◯ Refused to participate in questionnaire and HIV/TB Tests<br>◯ Agreed to both survey and HIV/TB Tests
DO NOT CONTINUE, THEY HAVE REFUSED STUDY PROCEDURES!!!!	
Please select the pack number of the coupons being issued to the participant. You can type the number and then select the correct option from the drop-down list	______________________
Date and time of interview	______________________
Unique identification code:<br>4 letters first name<br>4 letters surname<br>YOB<br>4 letters mother's name	______________________
Primary contact phone number	______________________

Demographic

DEMOGRAPHICS

What suburb do you live in? By live in I mean where do you normally sleep, for at least 3 nights/days of the week?

- ◯ Adamayview
- ◯ Alabama
- ◯ Boetrand
- ◯ Doringkruin
- ◯ Elandsheuwel
- ◯ Ellaton
- ◯ Flamwood
- ◯ Flimieda
- ◯ Freemanville
- ◯ Jouberton
- ◯ Kanana
- ◯ Klerksdorp Industrial
- ◯ La Hoff
- ◯ Manzilpark
- ◯ Meiringspark
- ◯ Neserhof
- ◯ Oudorp
- ◯ Roosheuwel
- ◯ Sakhrol
- ◯ Stilfontein
- ◯ Uraniaville
- ◯ Vaal Reef
- ◯ Wilkoppies
- ◯ Other (Klerksdorp)
- ◯ Other (outside Klerksdorp)

What racial group do you belong to?

◯ Black African ◯ Coloured
◯ White ◯ Indian/Asian
◯ Other

Where were you born?

◯ Gauteng ◯ KwaZulu Natal
◯ Western Cape ◯ Eastern Cape
◯ Northern Cape ◯ Free State
◯ Mpumalanga ◯ North West
◯ Limpopo ◯ Zimbabwe
◯ Mozambique ◯ Malawi
◯ Botswana ◯ Angola
◯ Namibia ◯ Zambia
◯ Lesotho ◯ Swaziland
◯ Democratic Republic of Congo
◯ Other

When did you arrive in South Africa?

____

When did you come to live in Klerksdorp?

____

What is your home language?

◯ isiZulu ◯ Ndebele ◯ isiXhosa ◯ Northern Sotho ◯ Sesotho ◯ Setswana ◯ Siswati ◯ Tshivenda ◯ Xitsonga ◯ English ◯ Afrikaans ◯ Other

Did you ever go to school? If so, what is the highest grade you have ever successfully achieved (passed)?

◯ No Schooling ◯ Primary School (Incomplete) ◯ Primary School (Completed) ◯ High School (Incomplete) ◯ High School (Completed) ◯ Post School Qualification

Have either of your parents died?

◯ Neither ◯ Mother ◯ Father ◯ Mother and father ◯ I do not know

How old were you when your mother died? ______________________________

How many living children do you have? ______________________________

How many of these children are under the age of 12? ______________________________

Do any of your children currently live with you? ◯ No ◯ Yes

What is the age of the youngest child who lives with you? ______________________________

How many people including yourself live in your house (the place where you stay)? ______________________________

How many rooms are in the house that you live in (ALL ROOMS)? ______________________________

	Often	Sometimes	Seldom	Never
Do the people in your home (including you) go without food?	◯	◯	◯	◯
Do the people in your home (including you) eat meat?	◯	◯	◯	◯

If a person in your home (including you) became very ill and R100 was needed for the treatment or medicine, how would it be to find the money?

◯ Very easy ◯ Easy ◯ Quite difficult ◯ Very difficult

In the past month, how many people (INCLUDING YOURSELF) have relied on your earnings/money for food, shelter or school fees? ______________________________

No	Yes

Selling sex is my primary source of income	◯	◯
I am employed by a company or person	◯	◯
I am unemployed	◯	◯
I receive a government grant such as a pension or child support grant	◯	◯
I sell/trade goods such as vegetables or clothes to earn money	◯	◯

In the last week, where did you normally sleep?

◯ Flat ◯ Hostel ◯ Tavern back room ◯ At home with my family ◯ A private house with other sex workers ◯ A private house with no other sex workers ◯ A brothel ◯ Abandoned building ◯ On the streets/nowhere in particular/constantly on the move ◯ Hotel ◯ Other

---

How many years have you been a sex worker for? __________________________________

How old were you when you first sold sex? __________________________________

Under what circumstances did you first sell sex? Select the one that is most close to what happened to you.

◯ No income to buy food or for shelter/kicked out
◯ Nowhere to stay, have nothing from being deserted, becoming an orphan or a widow/divorcee
◯ To add to the family income
◯ To pay for medical costs in the family or for self
◯ To earn fast money or meet personal expenses
◯ Tricked into going to a brothel
◯ Forced by family
◯ Forced by boyfriend
◯ Had to pay back someone through sexual favours
◯ Forced by stranger
◯ Forced by friend(s)
◯ Teenage pregnancy
◯ Other

Where do you normally pick up your client?

☐ Tavern ☐ Taxi rank ☐ Truck stop ☐ Prison ☐ Referral ☐ Street ☐ Hostel
☐ Hostel taverns ☐ Internet ☐ Newpaper/magazine avertisements ☐ Brothel ☐ Strip/dance cub
☐ Police ☐ Security ☐ Hair salon ☐ Pick-up (road/street corner) ☐ Mine ☐ Hotel
☐ Other

Pregnancy

PREGNANCY

How old were you when you first had sex?

____

Which of the following statements most closely describes your experiences the first time you had sexual intercourse?

○ I was willing ○ I was persuaded
○ I was tricked ○ I was forced
○ I was raped

Are you currently doing something or using any method to delay or avoid getting pregnant?

○ No ○ Yes

Which method are you using?

☐ The injection ☐ The pill
☐ Condoms ☐ The loop (IUD)
☐ Implant ☐ Herbal or other traditional medicines ☐ Spermicide
☐ Withdrawal before sperming/ejaculation
☐ Other

Have you ever been pregnant?

○ No ○ Yes

How old were you when you first became pregant

____

How many children have you given birth to ?

____

Have you ever been given PMTCT during ANY of your pregnancies? (medicine to prevent the baby getting HIV during pregnancy, birth or breastfeeding)

○ Yes
○ No

Are you pregnant now?

○ No ○ Yes ○ Unsure

How many months pregnant are you?

____

For your most recent pregnancy, did you take PMTCT during this pregnancy or birth

○ No, negative
○ Only at birth
○ During pregnancy and birth
(MOST RECENT INCLUDES CURRENT PREGNANCY)

Did you take ART while you were breastfeeding

○ No
○ Yes

After the pregnancy/breast feeding, did you continue taking treatment?

○ ART continued after pregnancy/breastfeeding ○ Stopped PMTCT but reinitiated ART later
○ Stopped PMTCT and has not taken ART since

Have you ever had a termination of pregnancy/abortion?

○ No ○ Yes ○

In your lifetime, how many abortions have you successfully had?

____

Have you ever experienced a miscarraige (not an abortion)

◯ Yes
◯ No

In your lifetime, how many miscarriages have you had?

____

In your lifetime, how many pregnancies have you had?

____

Have any of your children died after birth (this could be immediately afterwards or ever)?

◯ No ◯ Yes

How many of your children have died?

____
(IF >4, include comment and tell Programme Manager)

How old was your child when he/she died? (first child death)

◯ still birth ◯ from birth-28 days (4 weeks) ◯ 29 days - 12 months ◯ 1-4 years ◯ 5-12 years
◯ 13-18 years ◯ >18 years

How old was your child when he/she died? (second child death)

◯ still birth
◯ from birth-28 days (4 weeks)
◯ 29 days - 12 months
◯ 1-4 years
◯ 5-12 years
◯ 13-18 years
◯ >18 years

How old was your child when he/she died? (third child death)

◯ still birth
◯ from birth-28 days (4 weeks)
◯ 29 days - 12 months
◯ 1-4 years
◯ 5-12 years
◯ 13-18 years
◯ >18 years

How old was your child when he/she died? (fourth child death)

◯ still birth
◯ from birth-28 days (4 weeks)
◯ 29 days - 12 months
◯ 1-4 years
◯ 5-12 years
◯ 13-18 years
◯ >18 years

Hiv Knowledge

HIV KNOWLEDGE	
Have you previously tested for HIV?	◯ No ◯ Yes
Where was this test done?	◯ I can't remember ◯ Local clinic ◯ Hospital ◯ Other
What was your test result?	◯ I do not know ◯ Negative ◯ Positive ◯ Indeterminate
As far as you know today, what is your HIV status?	◯ Negative (no HIV) ◯ Positive (living with HIV)
If positive, when were you first diagnosed with HIV?	______________________ (if month and day unknown, insert 01-01-[YEAR])
Have you told your partner that you are living with HIV?	◯ No ◯ Yes ◯ I do not have a partner
When did you tell your partner that you were living with HIV?	______________________
Do you know your partner's HIV status?	◯ Unknown ◯ Negative ◯ Positive
Is your partner on treatment?	◯ No ◯ Yes ◯ Unsure
In the past 3 months, have you disclosed to any paying client what your HIV status is?	◯ Yes ◯ No
In the past 3 months, have you disclosed to any other women who sell sex, what your status is?	◯ Yes ◯ No
Are you on treatment?	◯ Never taken treatment ◯ On treatment always ◯ Stopped taking treatment (IF "YES" TO PMTCT, THEN HAS TAKEN TREATMENT)
When was the first time that you ever started taking treatment?	______________________
Have you ever had to reinitiate your treatment	◯ No ◯ Yes
In the past week how many times have you taken your treatment?	◯ All 7 days ◯ 5-6 days ◯ 3-4 days ◯ 1-2 days ◯ Not at all
Please rate your adherence to treatment in the past 4 week.	Non adherent — 100% adherence *(Place a mark on the scale above)*

[SHOW FDC CHART]<br>Are any of the following your ART medication?	◯ FDC - TDF+FTC+EFV<br>◯ FDC - TDF+FTC +(another)<br>◯ FDC - AZT+3TC +(another)<br>◯ FDC - ABC+3TC+ (another)<br>◯ None shown here
[SHOW SDF CHART]<br>In addition to the drugs you selected above, which other 1 ART drug are you taking?	◯ SDF - EFV<br>◯ SDF - LPV/r<br>◯ SDF - D4T<br>◯ SDF - ABC<br>◯ SDF - NVP<br>◯ SDF - AZT<br>◯ SDF - DDI<br>◯ SDF - 3TC<br>◯ Third Line (Other)<br>◯ Unsure
Which of the following 3 drugs are you taking?	☐ SDF - EFV<br>☐ SDF - LPV/r<br>☐ SDF - D4T<br>☐ SDF - ABC<br>☐ SDF - NVP<br>☐ SDF - AZT<br>☐ SDF - DDI<br>☐ SDF - 3TC<br>☐ Third Line (Other)<br>☐ Unsure<br>(3 CHOICES ONLY!!)
Other drug not on chart, please list names here if known	__________________________
What clinic do you go to for your treatment?	__________________________
What town & province is that clinic in?	◯ Gauteng<br>◯ North West<br>◯ Northern Cape<br>◯ Western Cape<br>◯ Eastern Cape<br>◯ KZN<br>◯ Limpopo<br>◯ Mpumalanga<br>◯ Free State<br>◯ Another country<br>◯ Unsure
Have you previously taken a different treatment / ART	◯ no<br>◯ yes
[SHOW FDC CHART]<br>Were you on any of the following medications?	◯ FDC - TDF+FTC+EFV<br>◯ FDC - TDF+FTC +(another)<br>◯ FDC - AZT+3TC +(another)<br>◯ FDC - ABC+3TC+ (another)<br>◯ None shown here

[SHOW SDF CHART]<br>In addition to the drugs you selected above, which other ART drug were you taking?	◯ SDF - EFV<br>◯ SDF - LPV/r<br>◯ SDF - D4T<br>◯ SDF - ABC<br>◯ SDF - NVP<br>◯ SDF - AZT<br>◯ SDF - DDI<br>◯ SDF - 3TC<br>◯ Third Line (Other)
Which of the following 3 drugs were you taking?	☐ SDF - EFV<br>☐ SDF - LPV/r<br>☐ SDF - D4T<br>☐ SDF - ABC<br>☐ SDF - NVP<br>☐ SDF - AZT<br>☐ SDF - DDI<br>☐ SDF - 3TC<br>☐ Third Line (Other)<br>☐ Unsure<br>(3 CHOICES ONLY!!)
Other drug not on chart, please list names here if known. [IF NAME UNKNOWN type 999]	________________________
How many different clinics have you taken ART from?	________________________
Have you ever taken a course of PEP (post exposure prophylaxis), so you were negative and were exposed to HIV, and the took medicine to stop you becoming infected (for example: condom bust, raped)	◯ No<br>◯ Yes<br>◯ unsure
how many times have you been given a course of PEP to take?	________________________
The most recent time that you took PEP, how many days did you take the PEP course for? (1-30 days)	________________________<br>(IF unsure how long took for, assist to calculate. IF remains unsure, enter 999)
Have you ever for any reason what-so-ever had a gap or interruption in taking your ART? [including any already mentioned]	◯ No<br>◯ Yes<br>(PMTCT interruption/single dose NVP = treatment interruption<br>IF answers "YES" but no previous treatment history, please go back and re-ask treatment history.)

The last time that you tested for HIV, what was the reason that you tested ?

◯ Peer educator encouraged me
◯ I wasn't feeling well ◯ Regular check-up
◯ prenatal checkup ◯ postpartum (after birth) checkup ◯ Condom burst
◯ Rape/sexual assault ◯ Concerned about my own health ◯ Partner is or was ill
◯ Partner is HIV positive
◯ Client was ill ◯ Client is HIV positive
◯ Family member has HIV
◯ Work requirement

Childhood

CHILDHOOD EXPERIENCES: Before you were 18 years of age, did you erxperience any of the following

	Never	Sometimes	Often	Very often
I did not have enough to eat	◯	◯	◯	◯
I lived in different households at different times	◯	◯	◯	◯
I saw or heard my mother beaten by her husband or boyfriend	◯	◯	◯	◯
I was told I was lazy or stupid or ugly by someone in my family	◯	◯	◯	◯
Someone touched my thighs, buttocks, breasts or genitals when I did not want him to, or made me touch his private parts when I did not want to	◯	◯	◯	◯
I was insulted or humiliated by someone in my family in front of other people	◯	◯	◯	◯

	Never	Sometimes	Often	Very often
I was beaten at home with a belt or stick or whip or something else which was hard	◯	◯	◯	◯
I had sex with a man who was more than 5 years older than me	◯	◯	◯	◯
One or both of my parents were too drunk to take care of me	◯	◯	◯	◯
I was beaten so hard at home that it left a mark or bruise	◯	◯	◯	◯
I spent time outside the home and none of the adults at home knew where I was	◯	◯	◯	◯
I had sex with someone who was not my boyfriend because I was threatened or frightened or forced	◯	◯	◯	◯

Did anyone that you lived with as a child, have sexual relationships with the purpose of helping to pay bills or engage in Ukhupanda? It could have been your mother/father, grannygrampa, aunt/uncle, sister/brother etc

◯ never
◯ sometimes
◯ often
◯ very often

Gendernorms

GENDER NORMS

Who are you sexually attracted to?
◯ Women ◯ Men ◯ Both men and women ◯ Neither men nor women ◯ Other ◯ I don't know

Are you currently married or do you have a partner? Are you living with this person?
◯ Currently married, living together ◯ Currently married, not living together ◯ Boyfriend/girlfriend, not married, living together ◯ Boyfriend/girlfriend, not married, not living together ◯ No current relationship

What gender is your current partner?
◯ Male ◯ Female ◯ Other

How long have you been with your current main partner? (years)
____

How old is your partner?
____

	No	Yes
Does your partner currently do anything to earn money?	◯	◯
Does your partner know that you sell sex?	◯	◯
Was your partner previously a paying client of yours (so you sold sex to them before you started dating)	◯	◯

Does your partner drink alcohol? How often?
◯ Never ◯ Every day/nearly every day ◯ Only at weekends ◯ A few times in a month ◯ Less than once a month ◯ I don't know

Have you ever fought/argued or had any other conflict over their drinking?
◯ No ◯ Yes

These questions are about your current or most recent intimate partner (non paying partner)

	Strongly agree	Agree	Disagree	Strongly disagree
When my partner wants sex they expect me to agree	◯	◯	◯	◯
If I asked my partner to use a condom they would get angry	◯	◯	◯	◯

My partner won't let me wear certain things	○	○	○	○
My partner has more to say than I do about important decisions that affect us	○	○	○	○

	Strongly agree	Agree	Disagree	Strongly disagree
My partner tells me who I can spend time with	○	○	○	○
When I wear things to look beautiful my partner thinks I may be trying to attract other men/women	○	○	○	○
My partner wants to know where I am all of the time	○	○	○	○
My partner likes to let me know they could have other girlfriends if they wanted to	○	○	○	○

	Strongly agree	Agree	Disagree	Strongly disagree
My partner would be angry if I used contraception without telling them	○	○	○	○
My partner and I are doing something to avoid getting pregnant	○	○	○	○
I think that a woman should obey her husband	○	○	○	○
I think that a man should have the final say in all family matters	○	○	○	○

	Strongly agree	Agree	Disagree	Strongly disagree
I think that a woman cannot refuse to have sex with her husband	○	○	○	○
I think that if a wife does something wrong her husband has the right to punish her	○	○	○	○
I think that if a woman doesn't physically fight back, it's not rape	○	○	○	○
I think that if a woman works she should give her money to her husband	○	○	○	○

	Strongly agree	Agree	Disagree	Strongly disagree
I think that a woman needs her husband's permission to do paid work	○	○	○	○

I think there is nothing a woman can do if her husband wants to have other girlfriends	○	○	○	○
I think that if a man has paid lobola (bride price) for his wife, he owns her	○	○	○	○
I think that if a man has paid lobola (bride price) for his wife, she must have sex when he wants it	○	○	○	○
I think that if a man beats you it shows that he loves you	○	○	○	○

Sex

SEX	
When was the last time you had sex (vaginal/anal/oral)?	__________________________________
The last time you had sex , was a condom used?	◯ Yes ◯ No
Was a condom used for every round (a round is until he cums/ejaculates/sperms)?	◯ Yes ◯ No
Who brought the condom, was it you or your partner?	◯ Self ◯ Partner ◯ Both
Did you experience any of the following with the condom?	◯ Condom breaking/bursting ◯ Condom slipping off ◯ Condom only put on halfway ◯ Condom removed during sex ◯ No problems, condoms worked perfectly
The last time you had sex, who was it with?	◯ Regter/main partner/vat 'n sit ◯ Other partner (affair) ◯ Client ◯ Ex partner ◯ One night stand
Have you used condoms with your MAIN PARTNER in the past month? How often would you say you used them?	◯ Always ◯ Often ◯ Sometimes ◯ Not used
How many MAIN (NON PAYING) PARTNERS have you had sex with in the last year?	__________________________________
How many OTHER NON-PAYING PARTNERS have you had sex with, or how many AFFAIRS have you had, during the past year?	__________________________________
How many one night stands (non-paying) have you had sex with during the past year?	__________________________________
Over the last year how often have you experienced the condom breaking or slipping off, or only put it on half way through, or have you taken it off and continued having sex?	◯ Every time used ◯ Often ◯ Sometimes ◯ Never ◯ Not used condoms
In the past 6 months have you experienced any of the following symptoms?	◯ Painful or burning vagina when urinating ◯ Sores or boils around vagina ◯ Itching vagina ◯ Abnormal/smelly vaginal discharge ◯ Pain in your lower stomach (not related to your menstrual period) ◯ None of these symptoms
Did you receive treatment for the symptoms you just mentioned?	◯ Yes ◯ No
Have you ever been diagnosed with a sexually transmitted infection such as gonorrhoea, syphilis, chlamydia, genital warts, or genital herpes?	◯ Yes ◯ No ◯ Unsure

Please select all of the different services that you offer	☐ Talking or companionship in person ☐ Massage ☐ Dinner ☐ Breast flashing ☐ Over-the-phone sex (talking) ☐ Whatsapp/sms sex ☐ Internet/email/online sex ☐ Normal/vaginal sex ☐ Anal sex ☐ Blow job (oral sex) ☐ Hand job ☐ Stripping or dancing ☐ Group sex ☐ Other
Please select the one service that is your most popular (that most clients ask for).	◯ Talking or companionship in person ◯ Massage ◯ Dinner ◯ Breast flashing ◯ Over-the-phone sex (talking) ◯ Whatsapp/sms sex ◯ Internet/email/online Sex ◯ Normal/vaginal sex ◯ Anal sex ◯ Blow job (oral sex) ◯ Hand job ◯ Stripping or dancing ◯ Group sex ◯ Other
In the past week, how many days did you have sex (oral, anal or vaginal/normal)with a client?	______________________
In the past month, how many days did you have sex (oral, anal or vaginal/normal) with a client?	______________________
How do your clients earn their money?	◯ Truck driving ◯ Miner ◯ farming ◯ theft/criminal activities ◯ other
How many one-time clients did you have sex with in the last working DAY?	______________________
How many one-time clients did you have sex with in the past WEEK?	______________________
The last time you had sex with one-time client did you go with a condom?	◯ Without condom ◯ Male condom ◯ Female condom ◯ Male and female condom
Do you always go with a condom with your one-time clients?	◯ Always ◯ Often ◯ Sometimes ◯ Never
How many of your regular clients did you have sex with in the last working DAY?	______________________
How many of your regular clients did you have sex with in the past WEEK?	______________________
The last time you had sex with a regular client did you go with a condom?	◯ Without condom ◯ Male condom ◯ Female condom ◯ Male and female condom

Has there been any occasion in the past month when either a regular or a one-time client went without a condom for any reason when you were having sex (so no condom was used at all)?	◯ Yes ◯ No ◯ Refused answer
Do you always work in the same place?	◯ Yes ◯ No
How many different places did you practise sex work in the past 6 months? (different taverns and different street corners, etc)	__________________________________
Have you ever worked as a sex worker in another province in South Africa?	◯ Yes ◯ No
Have you ever worked as a sex worker in another country (not South Africa)?	◯ Yes ◯ No
How much did you earn the last day that you sold sex?	__________________________________
How much did you earn the last week that you sold sex?	__________________________________
What is the MAXIMUM (MOST) amount that you earned in ONE DAY during this last month?	__________________________________
What is the MINIMUM (LEAST) amount that you earned in ONE DAY during this last month?	__________________________________
In the last month have you used anything to dry, clean or tighten your vagina before having sex? How often have you done this?	◯ Everytime ◯ Sometimes<br>◯ Once ◯ Never
What have you used?	☐ Soap ☐ Herbs/snuff<br>☐ Ice/water ☐ Zamlandela/tiger balm<br>☐ Alone stone ☐ Brown paper<br>☐ Ming balls ☐ Other
In the last month have you used anything to lubricate your vagina before having sex? How often have you done this?	◯ Everytime ◯ Sometimes<br>◯ Once ◯ Never
What have you used?	☐ water based lubricant<br>☐ Soap ☐ Spit ☐ Vaseline<br>☐ Baby Oil ☐ Other
In the last month have you used anything to hide that you were bleeding/mensruating, while having sex? How often have you done this?	◯ Everytime ◯ Sometimes<br>◯ Once ◯ Never

What have you used?	☐ Tampon ☐ Kitchen sponge<br>☐ Cloth ☐ Cotton wool<br>☐ Toilet paper ☐ Nacosa condom<br>☐ Brown paper ☐ Other
In the last 6 month, have you had any interactions with the police, IF so, how many times have you interacted with them?	______________________
Overall, of all of these interactions in the past 6 months, how many were negative interactions? E.G. bribed for sex, threatenned, violence used, harrased, verbally assaulted	______________________
Overall, how many of these interactions were positive? E.G. you received the service you needed in a respectful way, the arrest was done without violence and was legal	______________________

DISCRMINATION

	Never	Once	A few times	Often
Within the past year have you been verbally insulted, harassed or threatened because you are a sex worker?	◯	◯	◯	◯
Within the past year have you been beaten or threatened with beating because you are a sex worker?	◯	◯	◯	◯
Within the past year have you experienced sexual abuse because you are a sex worker?	◯	◯	◯	◯
Within the past year have you been denied health services because you are a sex worker?	◯	◯	◯	◯
Within the past year have you been denied police assistance/services because you are a sex worker?	◯	◯	◯	◯

Internalised Stigma

	Strongly agree	Agree	Disagree	Strongly disagree
Within the past year I have felt ashamed because I am a sex worker?	◯	◯	◯	◯
Within the past year I have felt guilty because I am a sex worker?	◯	◯	◯	◯

Within the past year I have blamed myself because I am a sex worker?	○	○	○	○
Within the past year I have felt that I am not worth anything because I am a sex worker?"	○	○	○	○

	Strongly agree	Agree	Disagree	Strongly disagree
Within the past year I have felt suicidal because I am a sex worker?	○	○	○	○
Within the past year I have isolated myself from friends and family because of being a sex worker?"	○	○	○	○
Within the past year I have avoided going to the clinic/hospital because of being a sex worker?"	○	○	○	○
Within the past year I have avoided going to the police because of being a sex worker?"	○	○	○	○

Violence

ALL VIOLENCE BY PERPS

Please ask the question and then read off each perpetrator type for a response. Mark if "Yes". If No perp category committed that question, then mark "None"

	INTIMATE PARTNER	CLIENT	POLICE	FAMILY	OTHER KNOWN	OTHER UNKNOWN	NONE
Within the past year did any partner insult you or make you feel bad about yourself?	☐	☐	☐	☐	☐	☐	☐
Within the past year did any partner make a show of you, belittle you, or humiliate you in front of other people?	☐	☐	☐	☐	☐	☐	☐
Within the past year did any partner do things to scare or intimidate you on purpose, for example by the way he looked at you, by yelling or smashing things?	☐	☐	☐	☐	☐	☐	☐
Within the past year did any partner threaten to hurt you?	☐	☐	☐	☐	☐	☐	☐
More than a year ago did any partner do any of these things to you?	☐	☐	☐	☐	☐	☐	☐

	INTIMATE PARTNER	CLIENT	POLICE	FAMILY	OTHER KNOWN	OTHER UNKNOWN	NONE
Within the past year did any partner slap you, push you or throw something at you which could hurt you?	☐	☐	☐	☐	☐	☐	☐
Within the past year did any partner hit you with a fist or with something else (such as a beer bottle, stick or belt) which could hurt you?	☐	☐	☐	☐	☐	☐	☐
Within the past year did any partner kick, drag, beat, choke or burn you?	☐	☐	☐	☐	☐	☐	☐
Within the past year did any partner threaten to use or actually use a gun, knife or other weapon against you?	☐	☐	☐	☐	☐	☐	☐

	INTIMATE PARTNER	CLIENT	POLICE	FAMILY	OTHER KNOWN	OTHER UNKNOWN	NONE
More than a year ago did any partner physically hurt you in any of these ways?	☐	☐	☐	☐	☐	☐	☐
Within the past year did any partner physically force you to have sex (vaginal/anal/oral) when you did not want to?	☐	☐	☐	☐	☐	☐	☐
Within the past year did you have sex (vaginal/anal/oral) with any partner when you did not want to because you were afraid of what he might do?	☐	☐	☐	☐	☐	☐	☐
More than a year ago did any partner force you to have sex (vaginal/anal/oral) when you did not want to?	☐	☐	☐	☐	☐	☐	☐

How many times has this (forced/fear sex/ rape) happened to you in the past 12 months

____
(IF ANY RAPE REPORTED, ENSURE PREVOIUS FORCED/FEAR SEX QUESTIONS ARE NOTED!)

How many times has this (forced/fear sex/ rape) happened to you in your lifetime?

____
(IF ANY RAPE REPORTED, ENSURE PREVOIUS FORCED/FEAR SEX QUESTIONS ARE NOTED!)

When was the most recent time you were made to have sex(vaginal/anal/oral) with a partner when you did not want it?

____

Have you ever experienced gang rape/streamlining/jackrolling

◯ Yes ◯ No

How often has this happened?

____

Mental Health

MENTAL HEALTH

	Rarely or none of the time	Some of or a little of the time (1-2 days)	A moderate/medium amount of time (2-4 days)	Most of the time (5-7 days)
During the past week I was worried by things that usually don't worry me	○	○	○	○
During the past week I had trouble keeping my mind on what I was doing	○	○	○	○
During the past week I felt depressed (very sad)	○	○	○	○
During the past week I felt that everything I did was an effort	○	○	○	○
During the past week I felt hopeful about the future	○	○	○	○

	Rarely or none of the time	Some of or a little of the time (1-2 days)	A moderate/medium amount of time (2-4 days)	Most of the time (5-7 days)
During the past week I felt fearful	○	○	○	○
During the past week my sleep was restless (woke up in the night/did not sleep well/had trouble falling asleep)	○	○	○	○
During the past week I was happy	○	○	○	○
During the past week I felt lonely	○	○	○	○
During the past week I could not get 'going'	○	○	○	○

Now I want to ask you a question about the past month. Has the thought of ending your life been on your mind? ○ No ○ Yes

Think of a time in your life when you have feared for your life, or been very traumatized. Now, in the past 2 weeks, have you felt any of the following?

	Not at all	Rarely	Sometimes	Most of the time
Do you have recurrent thoughts or memories of the event?	○	○	○	○
Do you have feelings as though the event is happening again?	○	○	○	○

Do you have recurrent nightmares about the event?	◯	◯	◯	◯
Do you have sudden emotional or physical reactions when reminded of the event?	◯	◯	◯	◯

	Not at all	Rarely	Sometimes	Most of the time
Do you avoid activities that remind you of the event?	◯	◯	◯	◯
Do you avoid thoughts or feelings associated with the event, so you try not to feel or think about anything that reminds you of it?	◯	◯	◯	◯
Do you feel jumpy, getting a fright easily?	◯	◯	◯	◯
Do you feel on guard, ready to fight to protect yourself?	◯	◯	◯	◯

Have you ever tried to kill yourself, attempted suicide? ◯ Yes ◯ No

Audit

SMOKING

Has the participant ever smoked tobacco?	◯ Yes, current smoker<br>◯ Yes, former smoker (used to smoke but not any more)<br>◯ No, never smoked
Which of the following has the participant ever smoked regularly	☐ Manufactured cigarette<br>☐ Handrolled cigarette<br>☐ Hookah/Hubbly Bubbly
How many manufactured cigarettes smoked per week?	__________________________________
How many hand rolled cigarettes smoked per week?	__________________________________
How many Hookah/Hubbly Bubbly smoked per week?	__________________________________
Has the participant ever used Snuff	◯ Yes, current snuff user<br>◯ Yes, former snuff user (used to use snuff but not any more)<br>◯ No, never used snuff
In the past 30 days, did anyone smoke indoors in areas where you work/live?	◯ Yes<br>◯ No
How often does anyone smoke indoors in areas where you work/live	◯ Daily<br>◯ Weekly<br>◯ Monthly<br>◯ Dont Know

ALCOHOL & DRUG USE

Have you drunk alcohol in the past 12 months?	◯ Yes ◯ No
How often do you have a drink containing alcohol?	◯ Never ◯ Monthly or less<br>◯ 2-4 times per month<br>◯ 2-3 times per week ◯ More than 4 times per week
How many drinks containing alcohol do you have on a typical day when you are drinking?	◯ 0 ◯ 1 or 2 ◯ 3 or 4<br>◯ 5 or 6 ◯ 7 to 9 ◯ 10 or more
How much is a typical drink (one drink, a beer or a glass of wine etc)	◯ No drink ◯ 250 mls or less (small beer or 1 glass of wine) ◯ 440mls<br>◯ 500mls ◯ 750 mls (bumpie)<br>◯ 1 litre ◯ 2 litres ◯ 5 litres (big box wine)

	Never	Less than monthly	Monthly	Weekly	Daily or almost daily
How often do you have six or more drinks on one occasion?	◯	◯	◯	◯	◯
How often during the past year have you found that you were not able to stop drinking once you started?	◯	◯	◯	◯	◯
How often during the past year did you find that you need a drink the next morning to get you going after a heavy drinking session?	◯	◯	◯	◯	◯
How often in the past year have you failed to do what was normally expected from you because of drinking?	◯	◯	◯	◯	◯
How often during the last year have you had a feeling of guilt or regret after drinking?	◯	◯	◯	◯	◯
How often during the last year have you been unable to remember what happened the night before because of drinking?	◯	◯	◯	◯	◯

Have you or someone else been injured as a result of drinking? ◯ No ◯ Yes but not in the last year ◯ Yes, during the last year

Has a relative or friend or health worker been concerned about your drinking or suggested you cut down? ◯ No ◯ Yes

Have you ever quarrelled with any of your partners about your drinking? ◯ No ◯ Yes

In the last 12 months how many times have you used drugs to make you high or have a good time? ◯ Never ◯ Once ◯ More than once

	Never	Once	Sometimes	Often
Within the PAST MONTH how often have you taken dagga?	◯	◯	◯	◯
Within the PAST MONTH how often have you taken mandrax ?	◯	◯	◯	◯
Within the PAST MONTH how often have you smoked nyaope or whoonga?	◯	◯	◯	◯
Within the PAST MONTH how often have you taken cough mixture when not sick?	◯	◯	◯	◯

Within the PAST MONTH how often have you taken pain killers when not sick?	○	○	○	○
Within the PAST MONTH how often have you taken Mg (ecstasy)?	○	○	○	○
Within the PAST MONTH how often have you taken dasha (crystal meth/tik)?	○	○	○	○
Within the PAST MONTH how often have you taken glass/rock/Ndanda?	○	○	○	○
Within the PAST MONTH how often have you taken heroin/ thai white?	○	○	○	○
Within the PAST MONTH how often have you done bluetooth?	○	○	○	○
Within the PATH MONTH how often have you taken cocaine	○	○	○	○

Tb Symptoms

TB SYMPTOMS

I am going to read you a list of possible symptoms of TB. Please think about the past few month. On how many days in the past month have you had the following symptoms? Please record the exact number of days:
none = 0
1 week = 7 days
1 month = 30 days
2 months = 60 days
3 months = 180 days

For how many days have you had a productive cough? ____________________

For how many days have you had a bloody or blood-stained cough? ____________________

For how many days have you expereinced any weight loss? ____________________

For how many days have you experienced any night sweats? ____________________

For how many days have you had a fever? ____________________

Have you ever previously been diagnosed with TB
- ◯ Yes
- ◯ No

What is the most recent date that you were diagnosed with TB? ____________________

If you took TB treatment, which applies to you? (MOST recent incident, please comment if >1 episode of TB reported)
- ◯ no treatment
- ◯ treatment completed, nurse/dr told you you were clear
- ◯ you stopped taking treatment on your own
- ◯ the Dr/nurse told you to stop taking it
- ◯ n/a

Are you currently on TB treatment?
- ◯ Yes
- ◯ No

In the past two weeks, did you take your TB treatment daily :
- ◯ Always
- ◯ Often
- ◯ Sometimes
- ◯ Never

Final

FINAL

I would like to thank you very much for helping us. We have talked about some very difficult things today. I appreciate the time you have taken. I realise that these questions may have been difficult for you to answer, but it is only by hearing about women's lives that we can really begin to understand them. We really appreciate your openness with us. Most women have difficult times in their lives and it is good to share them and remember we did not bring them upon ourselves. We really appreciate your participation in this study. By sharing this personal information with us and attending the peer talks and creative spaces you are helping us with our research and that will ultimately help many other sex workers in the country.

Between October - December 2017, did you go to the TB/HIV Care Association Clinic MOBILE CLINIC to do an HIV test OR ART Initiation OR STI treatment?	◯ Yes ◯ No
If you needed to go to the clinic, what clinic hours would suit you?	☐ Day ☐ Night
What 3 days of the week most suit you to go to the clinic?	☐ Monday ☐ Tuesday<br>☐ Wednesday ☐ Thursday<br>☐ Friday ☐ Saturday<br>☐ Sunday
In an ideal situation, what are all the services that you would like to receive at the clinic (read all and mark those that apply)	☐ HIV-related services<br>☐ STI services<br>☐ Family planning<br>☐ Pap smears<br>☐ Mammograms<br>☐ Baby wellness<br>☐ PMTCT<br>☐ Childhood immunisations<br>☐ Substance abuse<br>☐ Mental health<br>☐ Counselling<br>☐ Social worker<br>☐ Help with ID/birth certificate/passport other identifying documents<br>☐ Help with access to social grants<br>☐ Help with opening a bank account<br>☐ Nutrition support<br>☐ Support groups<br>☐ General minor ailments<br>☐ Safe space unrelated to HIV and sex work<br>☐ Rape or post trauma care<br>☐ Legal assistance
Could we contact you for any future research studies which may either be related to or unrelated to this study?	◯ Yes ◯ No
End Time of Interview	______________________
This interview took ___minutes to complete	______________________

Hct Counsellor

Pre Counselling Received	◯ Yes ◯ No

Thank you for taking this survey with me. Now that we have completed the survey, I am going to to explain what an HIV test is and what the results mean. I will then test you and while we wait for the results we can discuss ways to reduce your risk. You have already signed consent to do the test, so we will not need to sign consent again now.

First Rapid Test	◯ Reactive (Positive) ◯ Non-Reactive (Negative) ◯ Indeterminate
Second Rapid Test	◯ Reactive (Positive) ◯ Non-Reactive (Negative) ◯ Indeterminate
Did you negotiate a risk reduction plan	◯ Yes ◯ No

Hiv Tb

TESTS DONE AT VISIT

Tests Done by nurse

☐ Confirmatory Elisa assay (NHLS) ☐ CD4 (NHLS) ☐ Viral Load (NHLS) ☐ Drug Resistance (NICD)
☐ Gene Xpert (NHLS) ☐ TB Sputum (NHLS) ☐ Urine dip stick

NICD Laboratory Bar Code ______________________

NHLS Laboratory Bar Code ______________________

NHLS Laboratory Bar Code ______________________

Time of sputum collection ______________________
(select NOW)

URINE TESTS

COTANINE (COT) CASETTE

C
◯ Line present
◯ Line absent

T
◯ Line present
◯ Line faint
◯ Line absent

6 PANEL - MET/COC/THC

C
◯ Line present
◯ Line absent

	Line present	Line faint	Line absent
MET	◯	◯	◯
COC	◯	◯	◯
THC	◯	◯	◯

6 PANEL - MOP/BZO

C
- ◯ Line present
- ◯ Line absent

	Line present	Line faint	Line absent
MOP	◯	◯	◯
BZO	◯	◯	◯

6 PANEL - AMP

C
- ◯ Line present
- ◯ Line absent

AMP
- ◯ Line present
- ◯ Line faint
- ◯ Line absent

METHAQUALONE (MQL) DIPSTIX

C
- ◯ Line present
- ◯ Line absent

T
- ◯ Line present
- ◯ Line faint
- ◯ Line absent

Coupons

valid mobile phone number	__________________________________
First 3 letters of your name, first 3 letters of your surname, year of birth, first 3 letters of your mother's name	__________________________________
Please confirm the pack number of the coupons being issued to the participant. You can type the number and then select the correct option from the drop-down list	__________________________________
Select coupons issued	☐ female 1<br>☐ female 2<br>☐ female 3<br>☐ male 1<br>☐ male 2<br>☐ male 3

FSW COUPONS

Please confirm coupon 1 Number	__________________________________
Please confirm coupon 2 Number	__________________________________
Please confirm coupon 3 NumbeR	__________________________________

CLIENT COUPONS

Please confirm coupon 1 Number	__________________________________
Please confirm coupon 2 Number	__________________________________
Please confirm coupon 3 Number	__________________________________

Results

Confirmatory Elisa Result	◯ Negative ◯ Positive
CD4 count result	__________
Viral Load result	__________
GeneXpert result	◯ M Tuberculosis detected<br>◯ M Tuberculosis not detected<br>◯ Not done ◯ Error
GeneXpert Rif result	◯ Rifampacin resistance detected<br>◯ Rifampacin resistance not detected<br>◯ Not done ◯ Negative/Not Seen<br>◯ Error

Resistance Mutations

Drug Resistance Tested SUCCESSFULLY	◯ Yes ◯ VL < =400 therefore not tested<br>◯ NO - HIV Negative ◯ NO - Sample not haemolysed ◯ NO - Sample lost in transit<br>◯ NO - Failed blood draw<br>◯ NO - Refused blood draw<br>◯ NO - Missing for other reason
Susceptible to all	◯ Yes ◯ No
A62V	◯ Yes ◯ No
A98G	◯ Yes ◯ No
D67N	◯ Yes ◯ No
E138A	◯ Yes ◯ No
F227L	◯ Yes ◯ No
G190A	◯ Yes ◯ No
H221Y	◯ Yes ◯ No
K101E	◯ Yes ◯ No
K101P	◯ Yes ◯ No
K103N	◯ Yes ◯ No
K219E	◯ Yes ◯ No

K65R	◯ Yes ◯ No
K70E	◯ Yes ◯ No
K70R	◯ Yes ◯ No
K70T	◯ Yes ◯ No
L90M	◯ Yes ◯ No
M184V	◯ Yes ◯ No
M41L	◯ Yes ◯ No
P225H	◯ Yes ◯ No
T215F	◯ Yes ◯ No
T74S	◯ Yes ◯ No
V106M	◯ Yes ◯ No
V108I	◯ Yes ◯ No
V179D	◯ Yes ◯ No
V179E	◯ Yes ◯ No
V75L	◯ Yes ◯ No
V75MI	◯ Yes ◯ No
Y181C	◯ Yes ◯ No
Y188L	◯ Yes ◯ No
Notified Jenny? URGENTLY	◯ Yes ◯ No

Tier.net

Participant located on Tier.net	◯ Yes<br>◯ No
Are you certain that this is the same person	◯ yes, absolutely certain<br>◯ fairly certain<br>◯ not sure it is them

CONFIRMATION THAT THIS IS THE SAME PERSON:

First 3 letters of name & first 3 letters of surname (AS IT IS ON TIER.NET)	__________________________________
Year of birth on Tier.net	__________________________________
Gender ON TIER	◯ male<br>◯ female

MOST RECENTLY AVAILABLE TIER.NET DATA

What data is available?	☐ HIV test<br>☐ CD4<br>☐ Viral load<br>☐ ART<br>☐ TB
Date of most recent clinic visit	__________________________________
Date of most recent HIV test	__________________________________
HIV test result	◯ hiv negative<br>◯ hiv positive<br>◯ HIV + BUT LOST TO FOLLOWUP!
Date of most recent CD4	__________________________________
CD4 result	__________________________________
Date of most recent VIRAL LOAD	__________________________________
Viral load result	__________________________________
Date of most recent ART REGIMENT	__________________________________

ART regiment	◯ FDC - TDF+FTC+EFV<br>◯ FDC - TDF+FTC +(another)<br>◯ FDC - AZT+3TC +(another)<br>◯ FDC - ABC+3TC+ (another)<br>◯ None shown here
Additional ART	◯ SDF - EFV<br>◯ SDF - LPV/r<br>◯ SDF - D4T<br>◯ SDF - ABC<br>◯ SDF - NVP<br>◯ SDF - AZT<br>◯ SDF - DDI<br>◯ SDF - 3TC<br>◯ Third Line (Other)<br>◯ None shown here
Date of most recent TB DIAGNOSIS	__________________________
GeneXpert result	◯ M Tuberculosis detected<br>◯ M Tuberculosis not detected<br>◯ Not done<br>◯ Error
GeneXpert Rif result	◯ Rifampacin resistance detected<br>◯ Rifampacin resistance not detected<br>◯ Not done<br>◯ Negative/Not Seen<br>◯ Error
