# Supplementary material for: Key risk factors for substance use among female sex workers in Soweto and Klerksdorp, South Africa: A cross-sectional study
Source: PLoS One. 2022 Jan 21;17(1):e0261855. doi: 10.1371/journal.pone.0261855 (PMC8782394; doi:10.1371/journal.pone.0261855)
Supplement: S1 Table — (PDF) [file pone.0261855.s002.pdf]

**VARIABLE NAME**	CRONBACH ALPHA	MEASURE	DETAILS
**Depression**	0.68	CES-D 10 item scale (44)	A 4-point Likert scale was used to rate ten items. Tallied CES-D scores over 9 were considered to indicate major depression symptoms (i.e. a 9/10 cut point). Questions included: during the past week I was worried by things that usually don't worry me?, and during the past week I felt I was just as good as other people?
**Post-Traumatic Stress**	0.90	A short Post Traumatic Stress Disorder (PTSD) inventory (PTSD-8)(44) based upon the Harvard Trauma Questionnaire	A 4-point Likert scale was used to rate eight items on whether participants were bothered by various symptoms directly linked to the three subscales as part of the DSM-IV PTSD criteria(44) (hypervigilance, intrusion, avoidance). Questions referred to having ‘recurrent thoughts or memories of the event’, and ‘feeling jumpy, get a fright easily’ when thinking about the event. Scores were summed, and scores >3 for any of the three subscales were indicative of PTSD.
**Childhood Trauma**	0.78	Childhood Trauma Questionnaire (short version) (CTQ) (22)	Measured four dimensions (neglect; and emotional, physical, and sexual use). (physical and emotional) and use (emotional, physical and sexual). 12 items.<br>Items were scored separately and if participants had any sign of

			use from at least one of the dimensions, it indicated some childhood trauma.
**Physical use by IP or non-IP (client [paying partners], police, family members, other men)**	NA	WHO violence against women adapted for non-partner violence against FSWs	Physical use was assessed using the following four items: Within the past year, were you: 1) slapped, pushed, something thrown?; 2) hit with a fist or other object?; 3) kicked, dragged, beaten, choked or burnt?; and/or 4) threatened by a partner who wanted to use or used a gun, knife or other weapon against you?<br><br>Two variables were created using these items (physical use by an IP and physical use by a non-IP) with responses being categorised into none vs. some if participants had experienced any of the use from the items.
**Sexual use by IP or non-IP (client [paying partners], police, family members, other men)**	N/A	WHO violence against women adapted for non-partner violence against FSWs	Three items asking about being physically forced, afraid or threatened to have sex.<br>Sexual use was determined using three items asking about having sex (vaginal/anal/oral) due to either being afraid or physically forced, and asking how many times has this (forced/fear sex/rape) happened in the past 12 months.

			Two variables were created using these items (sexual use by IP and sexual use by non-IP) with responses none vs. some (per above).
**Alcohol and Drug Use**	0.85<br><br>N/A	Alcohol Use Disorder Identification Test (AUDIT)-C scale(5) was adapted and used to show severe binge drinking.<br><br>Self-reported drug use. Cocaine and heroin were not consistently asked across both studies therefore were excluded in the analysis.	Measures binge drinking through four items: the 3 AUDIT-C questions (How often do you have a drink containing alcohol?, How many drinks containing alcohol do you have on a typical day when you are drinking?, How often do you have six or more drinks on one occasion?), and one added question on the volume of alcohol per drink (mL). The four items were summed into a score, with a cut-off of 10 used to indicate binge drinking.<br><br>Drug use asked about using locally relevant drugs within the past year (i.e.; dagga, mandrax, nyaope, cough mixture, ecstasy, tik, painkiller, rock).
