# Supplementary material for: Key risk factors for substance use among female sex workers in Soweto and Klerksdorp, South Africa: A cross-sectional study
Source: PLoS One. 2022 Jan 21;17(1):e0261855. doi: 10.1371/journal.pone.0261855 (PMC8782394; doi:10.1371/journal.pone.0261855)
Supplement: Attachment — Submitted filename: Comments.docx [file pone.0261855.s003.docx]

Overall, really interesting paper with really interesting findings. There is just one piece that concerns me is drug use in the past month for Klerksdorp population and drug use in the past year for the Soweto population. See my last comment for suggestions on how to address this. All other comments are minor revisions.

Line 116-117 The intro section is full of strong statistics about SU in FSW populations. It is a bit of a contradiction to say something has been infrequently studied, when you have presented several articles to support SU in FSW populations (Line 65-68, 70, 72,77). The sentence should read “despite the high prevalence of trauma and mental health, gaps exist when focusing on binge drinking and drug use (although I do think you provided evidence that there is no gap when focused on drug use). The focus of this study is to describe the prevalence of self-reported binge drinking, and associated risk factors” or something similar. I think just a simple reivision will address this

Line 124 “enroll” Small spelling error

Line 126-127 “The full methodology from the Soweto study has been extensively described previously (7)” Language could be a better. For example: “The methods from the Soweto study has been throughly described in previous publications (7)”

Keep language consistent. Sometimes you utilize “FSW” and sometimes “woman.” For consistency, choose one! (End of line 136).

 Line 137 “informal sex work was also paid for in beer” Do you have a citation for this?

Line 168 “Post enrollment” Another small spelling error.

Line 411-413 This is interesting and could also be a focus of future research. In what circumstances are FSW using both?

Line 450-452 The focus on HIV and ART feels a little out of place. I understand the big picture relationship, but how does this relate to the objective of the analysis which was to determine the prevalence and associated risks of binge drinking and drug use?  Other limitations: cross sectional design.

Line 455: Because the Kleksdorp population was only asked about past month drug use, was this adjusted to reflect what the past year use would be? This assumes that every month has similar patterns of drug use, which I think is a feasible assumption. This could also explain why drug use was higher in Soweto compared to Kerksdorp. I think this is a glaring problem that needs to be adjudicated in either the methods to explain how you adjusted for this discrepancy or in the results, where you use the one month report to predict one years use, using the assumption the the report of one month's use is reflective of use in all the other months. This may change your results.
